# Concurrent HHV-8-Associated Multicentric Castleman Disease and Kaposi Sarcoma in an HIV-Negative Patient: A Case Report

**DOI:** 10.3390/diagnostics16111622

**Published:** 2026-05-26

**Authors:** Alin-Marian Vasile, Raluca-Maria Closca, Marina Rakitovan, Maria Iordache, Flavia Zara

**Affiliations:** 1 Department of Pathology, Emergency City Hospital, 300254 Timisoara, Romania; 2 Department of Microscopic Morphology, University of Medicine and Pharmacy “Victor Babes”, 300041 Timisoara, Romania; 3 Oro–Maxillo–Facial Surgery Clinic, Emergency City Hospital, 300062 Timisoara, Romania; 4 First Department of Internal Medicine, University of Medicine and Pharmacy “Victor Babes”, 300041 Timisoara, Romania; 5 Department of Hematology, Emergency City Hospital, 300254 Timisoara, Romania

**Keywords:** Human Herpesvirus 8, Kaposi Sarcoma Herpesvirus, multicentric Castleman disease, Kaposi Sarcoma, rituximab, liposomal doxorubicin

## Abstract

**Background and Clinical Significance:** Human Herpesvirus 8-associated multicentric Castleman disease is a rare, lymphoproliferative disorder characterized by recurrent episodes of systemic inflammation. The disease is predominantly observed in human immunodeficiency virus-positive patients, but there is evidence of its occurrence in negative individuals. Its pathogenesis is driven by dysregulated cytokine activity, particularly interleukin 6. Additionally, these individuals have an increased risk of developing Kaposi Sarcoma, which may present simultaneously. **Case Presentation:** The current paper presents a case of a 58-year-old male patient admitted to the Hematology Department of the Emergency City Hospital in Timisoara, Romania, in October 2024, accusing fever, night sweats, palpitations, weight loss and general deterioration, approximately three months prior, with gradual progression. Clinical examination revealed bilateral lymphadenopathy in the cervical and inguinal regions. No cutaneous lesions were observed initially. Laboratory tests showed elevated inflammatory markers, pancytopenia, hypergammaglobulinemia and hyponatremia. HIV serology had negative results. CT imaging revealed extensive lymphadenopathy and splenomegaly. Further excisional biopsy of the inguinal and cervical lymphadenopathies was performed. Following the microscopic examination, the final diagnosis of Human Herpesvirus 8-associated multicentric Castleman disease concurrent with Kaposi Sarcoma was established. **Conclusions:** Human Herpesvirus 8-associated multicentric Castleman disease is predominantly observed in HIV-positive patients, but there is evidence of its occurrence in human immunodeficiency virus-negative individuals, presenting distinct epidemiological and pathological characteristics. Early and precise diagnosis is essential, as the disease can progress rapidly and may lead to severe or fatal outcomes.

## 1. Introduction

The term Castleman disease (CD) has been used to describe several different lymphoproliferative disorders that share a range of characteristic histopathological features. CD is classified into two main types: unicentric Castleman disease (UCD) and multicentric Castleman disease (MCD); MCD being further divided into idiopathic MCD (iMCD) and Human Herpesvirus 8-associated MCD (HHV-8-MCD) [1,2,3,4]. HHV-8, also referred to as Kaposi Sarcoma Herpesvirus (KSHV), is the etiological agent responsible for a range of conditions, including HHV-8-MCD, Kaposi Sarcoma (KS), primary effusion lymphoma (PEL) and a recently described interleukin 6 (IL-6)-related disease called KSHV inflammatory cytokine syndrome (KICS) [5,6,7,8,9].

HHV-8-MCD is a lymphoproliferative disorder marked by lymphadenopathy and inflammatory symptoms, with a fluctuating course, driven by excessive IL-6 production, while Kaposi Sarcoma is an angioproliferative disorder [10]. Since both diseases share a common etiological agent, they may occur simultaneously, particularly in individuals with underlying immunodeficiency [11].

Similar to other herpesviruses, HHV-8 undergoes two main phases of gene expression: a latent phase and a lytic phase [12,13,14]. During the latent phase, HHV-8 gene expression is highly restricted, with most of the expressed genes focused on promoting cell survival, preventing apoptosis and avoiding detection by the host immune system [15,16,17]. The virus can remain latent in various cell types, including endothelial cells and B-lymphocytes [18,19]. During this period, the infected patients usually remain asymptomatic.

After the activation, HHV-8 transitions into the lytic phase, where all viral genes are expressed and the host cell machinery is repurposed to produce and assemble new virions [20]. The interaction between the virus and the host cells triggers the expression of inflammatory proteins, including both human and viral interleukin IL-6 [21,22,23]. Dysregulation of inflammatory cytokines is considered to be the primary pathological mechanism in KSHV-MCD [24,25,26]. Latent and lytic genes very between HHV-8-related disorders, however, in the case of KSHV-MCD, infected plasmablasts in the affected lymph nodes often express lytic proteins [27,28,29]. The activation of the lytic phase is most commonly present in people co-infected with HIV, although some rare cases can occur in HIV-negative individuals. Intermittent reactivation of the lytic phase can also be triggered by several stimuli, including other infections, inflammation, or oxidative stress [30].

Radiologic findings in these cases are generally nonspecific. Computed tomography (CT) scans often demonstrate extensive lymphadenopathy affecting multiple nodal regions, accompanied by hepatosplenomegaly. These imaging features reflect the systemic involvement typical for the disease; however, they are not exclusive; correlation with clinical and pathological findings for accurate diagnosis being required. An excisional biopsy of the most accessible enlarged lymph node should be selected for histopathological confirmation [31].

## 2. Case Presentation

The current paper presents a case of a 58-year-old male patient admitted to the Hematology Department of the Emergency City Hospital in Timisoara, Romania in October 2024, accusing fever, nocturnal hyperhidrosis, palpitations, weight loss and general debilitation. The symptoms began approximately three months earlier and gradually worsened over time.

Upon clinical examination, bilateral enlargement of the inguinal lymph nodes was noted, measuring up to 2.5 cm in diameter, being of an elastic consistency, mobile and painless on palpation. Edema was observed in the lower extremities and abdominal wall. The vital signs values of the patient noted: a body temperature of 38.2 °C, heart rate of 110 bpm and blood pressure of 95/60 mmHg. Initially, no cutaneous lesions were present.

The patient’s laboratory tests revealed several abnormal findings, including anemia, thrombocytopenia, and leukopenia. Elevated inflammatory markers were also observed, such as C-reactive protein, along with serum hyponatremia, and even elevated procalcitonin levels (Table 1). Based on these clinical and laboratory findings, an initial presumptive diagnosis of infectious sepsis was established.

Empirical treatment with broad-spectrum antibiotics and antifungal medications was promptly initiated in an attempt to address the patient’s condition. Despite the administration of these treatments, the patient’s overall clinical status did not show any noticeable improvement. Furthermore, C-reactive protein levels remained significantly elevated. Fungal and bacteriological blood cultures and from a pharyngeal swab were performed, but no fungal elements nor bacterial growth were observed on the culture plates.

Following additional serologic testing, the patient tested negative for human immunodeficiency virus, negative for hepatitis B virus, and negative for hepatitis C virus. Additionally, blood IL-6 levels were measured, revealing elevated values of 122.7 pg/mL.

A sternal bone marrow aspirate revealed a normocellular marrow with representation of all hematopoietic lineages. Lymphocytes were noted at 6%, while plasma cells were significantly elevated at 20.5%. These plasma cells displayed variable nuclear morphology, with some exhibiting 1 to 3 nuclei. Megakaryocytes were present with thrombocytogenic activity. Perls’ staining showed absent iron deposits. These findings are indicative of iron deficiency and plasmocytic hyperplasia; nevertheless, further investigation was needed in order to exclude infectious or inflammatory conditions. A follow-up coxal bone marrow aspirate was found to be within normal parameters.

Regarding the patient’s peripheral lymphadenopathy, a thoracic, abdominal and pelvic computed tomography scan was performed. The CT scan revealed extensive lymphadenopathy in the inguinal, intra-abdominal, retroperitoneal and mediastinal regions, with the largest lymph node situated in the inguinal region and measuring up to 4.5 cm in diameter (Figure 1). Additional splenomegaly was observed.

Further excisional lymph node biopsy was performed from the inguinal region. The harvested tissue was fixed in 10% (*v*/*w*) neutral buffered formalin and was sent to the Department of Pathology of Timisoara Emergency City Hospital for a histopathological examination.

The macroscopic examination revealed an enlarged lymph node, measuring 3/1.8/1.3 cm, with a firm and elastic consistency and yellowish-brown external surface. On the cut surface the lymph node had a homogeneous yellowish-white appearance.

For diagnostic purposes, serial sections of 4 μm thickness were cut from the paraffin-embedded samples and stained using the standard Hematoxylin–Eosin (HE) method. The histopathological diagnosis was further complemented with immunohistochemical staining using antibodies such as: anti-CD31, anti-CD34, anti-D2-40, anti-HHV-8 Latency-Associated Nuclear Antigen 1 (HHV-8-LANA1), anti-kappa light chains, anti-lambda light chains, and anti-CD138.

Details of the antibodies employed for the immunohistochemical analyses are summarized in Table 2. The antibody panel as well as the immunohistochemistry reagents were obtained from Novocastra™ and Bond™, respectively (Leica Biosystems, New Castle, UK).

Microscopic examination using Hematoxylin–Eosin staining showed architectural distortion of the lymph node, with a proliferation of spindle-shaped cells forming slit-like vascular spaces and exhibiting moderate atypia. Morphological findings also included extravasated erythrocytes, intracellular hyaline globules, and scattered hemosiderin-laden macrophages. Immunohistochemical staining demonstrated positivity for CD31, CD34, and D2-40, while EBV showed negative reaction. Further, HHV-8 Latency-Associated Nuclear Antigen 1 (HHV-8-LANA1) staining identified Human Herpesvirus 8-infected cells within the spindle cell proliferation (Figure 2).

In addition to the previously noted changes, the lymph node exhibited vascular proliferation and hyalinization of vessel walls, atrophic germinal centers, characterized by the germinal center lymphocyte depletion, thickened mantle zones with lymphocytes arranged in layers—“onion ring” appearance, sclerotic vessels penetrating the follicles—“lollipop” appearance, some follicles showing blurring boundaries of the mantle zone and fusion between adjacent germinal centers—twinning appearance; the other germinal centers were hyperplastic. HHV-8-LANA1 staining identified numerous HHV-8 infected cells, located predominantly in the mantle zone of the follicles (Figure 3).

Additionally, scattered plasma cells were observed in the mantle zone, while sheets and nodules of plasma cells were present in the interfollicular areas. These cells exhibited medium to large mononuclear morphology with amphophilic cytoplasm, large nuclei and vesicular chromatin. The immunohistochemical profile demonstrated a polytypic plasma cell population, with an approximate 2:1 ratio, expressing both kappa (70%) and lambda (30%) light chains (Figure 4).

Based on the results of microscopic examination using Hematoxylin–Eosin staining, correlated with immunohistochemical findings, in the context of HHV-8 infection, the diagnosis of Human Herpesvirus 8-associated multicentric Castleman disease concomitant with Kaposi Sarcoma was established.

The patient received erythrocyte and platelet transfusions, resulting in hematologic improvement. Following the diagnosis, subsequent oncologic treatment with pegylated liposomal doxorubicin and rituximab was well tolerated, leading to clinical and biochemical remission (Table 3).

## 3. Discussion

In 1994, Chang et al. used representational difference analysis to isolate a unique genetic sequence of a previously unknown human gamma herpesvirus in tissues obtained from Kaposi Sarcoma lesions [32]. This virus with two given names, Human Herpesvirus 8 (HHV-8) and Kaposi’s Sarcoma Herpesvirus (KSHV), was quickly linked to several other pathologies. In 1995, only a year after its discovery, researchers first demonstrated its role in the etiology and pathophysiology of multicentric Castleman disease [14].

HHV-8 can establish a lifelong infection, remaining in a latent phase without causing clinically apparent symptoms in immunocompetent hosts [33,34,35,36]. Studies have demonstrated the presence of HHV-8 DNA in peripheral blood cells, saliva, and tonsillar swabs of some asymptomatic individuals, suggesting that the virus can maintain latency without inducing an overt disease [33,37,38]. A weakened immune system may allow the virus to activate, triggering its lytic phase and potentially causing related diseases [32]. The causes of immunodeficiency can be categorized as intrinsic (primary) or extrinsic (secondary). The most common factor associated with HHV-8 lytic phase activation is the co-infection with HIV, followed by immunosuppressive treatments [39].

In some rare cases, KSHV-MCD can occur in the absence of HIV infection or immunosuppressive therapy, with the mechanisms of the underlying HHV-8 activation remaining unclear. Other factors may contribute as well, such as other infection agents, inflammation, cigarette smoking, alcohol consumption, oxidative stress, or even the number of sexual partners [30,40,41]. A quick review of the literature reveals previously reported cases of HHV-8 activation in patients who appear otherwise immunocompetent [42,43]. Dossier et al. conducted a study on KSHV-MCD in HIV-negative individuals and found that among 18 HHV-8-positive patients, none showed evidence of immunosuppression [44]. Powles et al. suggest that MCD is not only associated but may also depend on some degree of immune system integrity to facilitate interaction with HHV-8 [45]. In our case, the patient exhibited no identifiable extrinsic cause of immunodeficiency, testing negative for HIV, HBV, and HCV, with no history of immunosuppressive treatment. Additionally, blood cultures for bacterial and fungal infections were negative.

In 2023, Romain Stemmler et al. conducted a study analyzing 80 patients with HIV–HHV-8+ MCD and compared their characteristics with 228 patients with HIV+ HHV-8+ MCD [46]. The baseline characteristics of both groups were largely similar. HIV-negative patients were diagnosed with MCD at an older age (median 67 vs. 43 years). Male predominance was also lower in the HIV-negative group (74% vs. 82%). Biological markers, such as serum CRP, gamma globulin levels, cytopenia, and blood HHV-8 DNA viral load, were comparable between both of the groups. However, MCD symptomatic episodes were generally less aggressive in HIV-negative patients, compared with those with detected HIV. The median overall survival for HIV-negative patients is 8.8 years, with a 5-year survival probability of 79.2%, closely resembling the outcomes seen in HIV-positive individuals.

Despite both being rare conditions, HHV-8-MCD and KS frequently occur simultaneously, suggesting that they may represent different tissue responses to the same pathological agent [47,48,49,50,51,52,53,54]. It is also common to find both HHV-8-MCD and KS coexisting within a single tissue sample [55].

According to Kikkeri N. Naresh et al., HHV-8-infected B-lymphoid cells (plasmablasts) help sustain the viral load in affected individuals, which may contribute to the formation of new KS lesions [55]. Since HHV-8 infection in MCD follows a “lytic” form, it serves as a continuous source of virions capable of spreading to nearby endothelial cells. Furthermore, endothelial cells within the capsule, trabeculae and hilum of the lymph node may be particularly vulnerable to HHV-8 infection, potentially undergoing transformation and forming KS tumorlets at these sites. Additionally, Romain Stemmler et al. reported a lower prevalence of KS lesions in HIV-negative patients with KSHV-MCD compared to those who were HIV-positive (34% vs. 52%) [46].

The initial clinical presentation of KS consists of purple macules or papules, which progressively transform into plaques or nodular lesions. The most commonly affected skin areas are the lower extremities, feet and the torso; nonetheless, it can sometimes extend to the upper extremities, hands, head, and neck area [56]. Although uncommon, KS can first manifest with lymphadenopathy preceding any skin lesions. Both cutaneous and nodal forms of KS have been reported in association with MCD [47,48,49,50,52,53]. As for the presented case, the patient was initially admitted with primary lymph node involvement of KS and he did not exhibit any skin lesions at the time of diagnosis.

A wide variety of cells are responsible for the production of IL-6, including T cells, B cells and endothelial cells. IL-6 is involved in the differentiation and proliferation of B lymphocytes and T lymphocytes, as well as in the production of acute-phase reactant proteins in the liver. Consequently, it contributes to the development of symptoms that are commonly observed in various inflammatory pathologies [57]. IL-6 also stimulates the liver to secrete the peptide hormone hepcidin, which further disrupts iron absorption and metabolism, contributing to the development of chronic anemia [58].

CD has three histological variants: hyaline vascular, plasma cell, and mixed. The plasma cell variant is generally associated with a more aggressive clinical course and reduced response to anti-IL-6 therapy compared to the hyaline vascular type, although different patterns may coexist or evolve within the same patient over time [59].

HHV-8-MCD most often resembles the plasma cell variant, but may include mixed histological features, often with the preservation of lymph node architecture [60]. HHV-8-infected plasmablasts are characterized as medium to large mononuclear cells with amphophilic cytoplasm, observed within the mantle zones of the nodal follicles or in the interfolicular zones. The infection with HHV-8 is confirmed by immunohistochemistry, which reveals expression of viral proteins such as latent nuclear antigen-1.

In the early stages of the disease, these plasma cells are more often polytypic, whereas in later stages they tend to become monotypic, frequently exhibiting IgM lambda light chain restriction. The reason behind the preferential expression of the lambda light chain in these cells remains further under investigation. Wang and his colleagues have summarized evidence from recent studies indicating that HHV-8-driven transcriptional activity may lead to preferential selection of lambda-expressing plasma cells [61]. These plasmablasts may occasionally cluster together, resulting in prominent aggregates, which may raise histologic concern for malignancy [62,63]. In such cases, the main differential diagnosis is HHV-8-positive diffuse large B-cell lymphoma. In earlier World Health Organization (WHO) classifications, small aggregates of plasmablasts were termed “microlymphomas”. However, the latest WHO revisions recognize that not all plasmablastic aggregates are clonal, explaining why only a subset progresses into lymphoma. The presented case is consistent with previous observations, as our patient exhibited a mixed histological pattern, characterized by vascular proliferation, hyalinization of vessel walls and the presence of plasma cell sheets and nodules. Although both light chains were expressed in the plasma cells, a slight restriction of lambda chains was noted, suggesting a shift towards monoclonality, which may explain the tendency of these cells to form aggregates in the interfollicular areas.

Plasma cell elevation was also noted in the bone marrow aspirate of our patient. These findings align with established scientific reports on MCD, where the bone marrow may be involved, often demonstrating bland plasmacytosis. In some cases, reticulin fibrosis or HHV-8-infected cells may also be identified [64].

Similar to our case, a subset of Kaposi Sarcoma cases exhibit absent or reduced CD31 expression. Although CD31 is a reliable marker in other vascular neoplasms, its poor staining in KS has been linked to HHV-8 infection and reflects the virus’s immune evasion mechanisms. One of the proteins encoded by the virus, known as K5, is responsible for downregulation of the CD31 receptor on infected endothelial cells [65]. K5 protein expression is strongly correlated with low levels of CD31, but not CD34 [66,67]. Supporting this, Nunes Rosado et al. assessed various vascular and lymphatic endothelial markers in KS diagnosis and found that CD31 was less sensitive than CD34 and D2-40, especially in nodular lesions [68].

Therapeutic approaches for HHV-8-MCD have included several cytotoxic chemotherapies commonly used for B-cell lymphomas, such as etoposide, vincristine, vinblastine, cyclophosphamide and doxorubicin. Regardless, outcomes have been poor, with a median overall survival of only 12 months [60]. The introduction of rituximab, a monoclonal antibody specifically targeting the CD20 antigen on B-cells, led to significant advancements in treatment response and patient survival.

Bower et al. reported that 21 previously untreated patients with symptomatic KSHV-MCD received rituximab (375 mg/m^2^) for four weekly doses, achieving rapid clinical remission and a 2-year overall survival of 95%, although KS progression occurred in over one-third of cases [69]. Similarly, the CastlemaB Trial included 24 patients with HIV-associated KSHV-MCD treated with the same rituximab regimen, reporting a 92% overall survival and sustained remission in most patients at one year, with frequent KS exacerbation in those with prior disease [70]. While most evidence derives from HIV-positive populations, rituximab has also shown efficacy in HIV-negative HHV-8-associated MCD [44].

To address KS worsening associated with rituximab, a prospective study evaluated rituximab combined with liposomal doxorubicin every three weeks for several cycles. This approach showed improved disease control, with limited progression and favorable survival outcomes [71].

In the presented case, due to the patient’s concurrent KS, a treatment regimen based on rituximab combined with liposomal doxorubicin was chosen. The therapy was well tolerated by the patient, achieving both clinical and biochemical remission.

This study has some limitations that should be acknowledged. Plasma HHV-8 DNA quantification was not routinely available for patients, preventing a comprehensive assessment of the relationship between viral load, viral reactivation, and inflammatory markers such as IL-6. As a result, we were unable to evaluate whether changes in cytokine levels correlated with the degree of HHV-8 viremia. Prospective studies with larger cohorts, standardized longitudinal sampling, and integrated viral and cytokine profiling are needed to better clarify the pathophysiological mechanisms underlying these observations.

Another limitation of the present study is the absence of EBER in situ hybridization analysis in the evaluated tissue samples. Although immunohistochemical staining for EBV was performed and yielded negative results, EBER ISH represents the gold standard method for detecting latent Epstein–Barr virus infection in tissue specimens and may provide higher sensitivity in identifying EBV-associated lymphoproliferative processes. The lack of EBER ISH testing reflects the retrospective nature of the study and the diagnostic protocols available at the time of case evaluation. Consequently, the possibility of an occult EBV-driven component cannot be entirely excluded.

## 4. Conclusions

Human Herpesvirus 8-associated multicentric Castleman disease is predominantly observed in HIV-positive patients; however, there is evidence of its occurrence in HIV-negative individuals, presenting distinct epidemiological and pathological characteristics. Given the nonspecific nature of the symptoms especially in HIV-negative patients, diagnosis requires a high level of suspicion on the part of the clinician, as the features overlap with those observed in various other inflammatory conditions. Accurate diagnosis requires the integration of clinical evaluation, imaging studies, laboratory investigations and histopathological examination.

Overall, reporting unusual cases of Human Herpesvirus 8-associated multicentric Castleman disease in HIV-negative patients contributes to the enrichment of the literature and helps deepen our understanding of this rare condition, its development, and its overall impact.

## Figures and Tables

**Figure 1:**
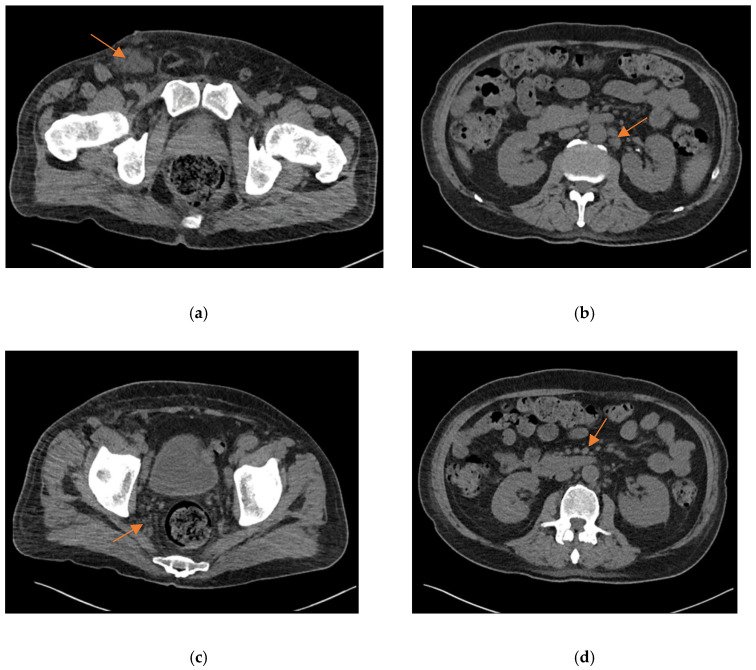
Computed tomography scan: (**a**) inguinal lymphadenopathy (arrow); (**b**) retroperitoneal lymphadenopathy (arrow); (**c**) pelvic region lymphadenopathy (arrow); (**d**) intra-abdominal lymphadenopathy (arrow).

**Figure 2:**
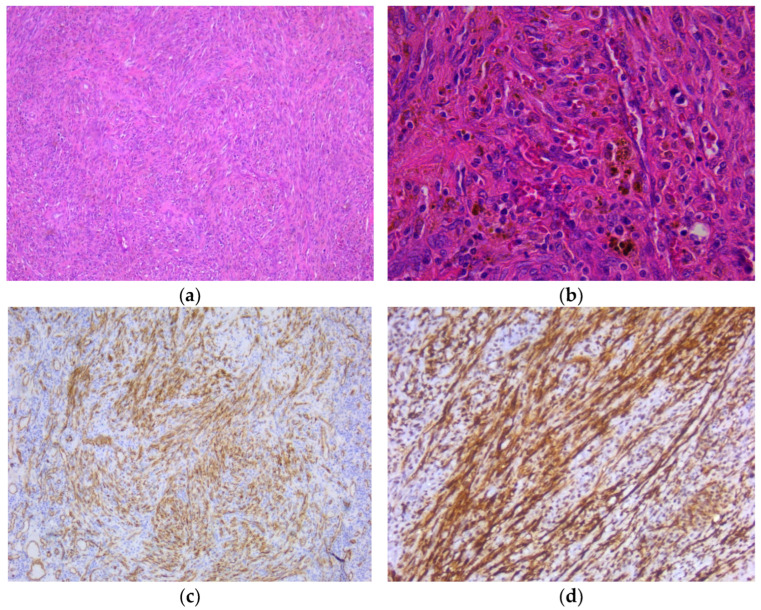

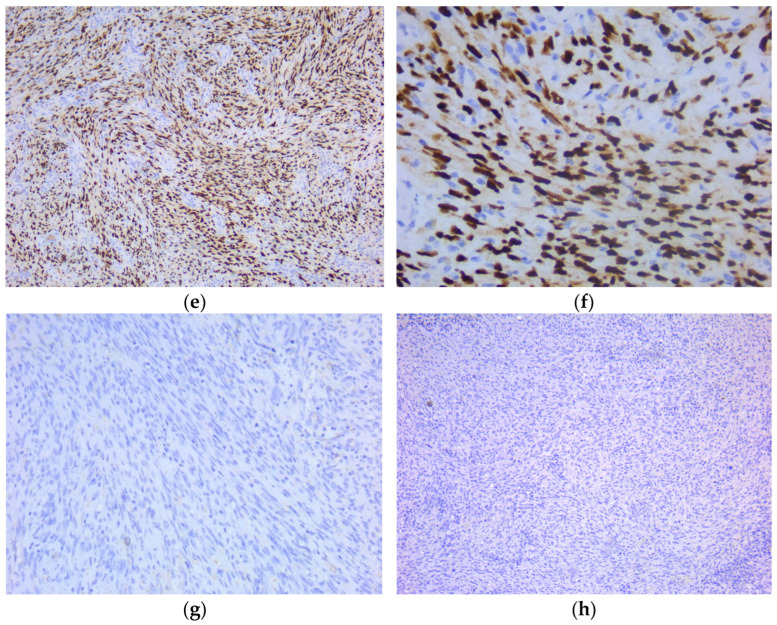
Microscopic aspects: (**a**) Hematoxylin–Eosin-stained slide showing spindle cell proliferation, ob. 10×; (**b**) Hematoxylin–Eosin-stained slide showing spindle cell proliferation, ob. 40×; (**c**) CD34, strong and diffuse positive reaction, ob. 20×; (**d**) D2-40, diffuse positive reaction, ob. 20×; (**e**,**f**) HHV-8-LANA1, intense nuclear staining, ob. 10× and ob. 40×. (**g**) CD31, negative reaction, ob. 10×; (**h**) EBV, negative reaction, ob. 20×.

**Figure 3:**
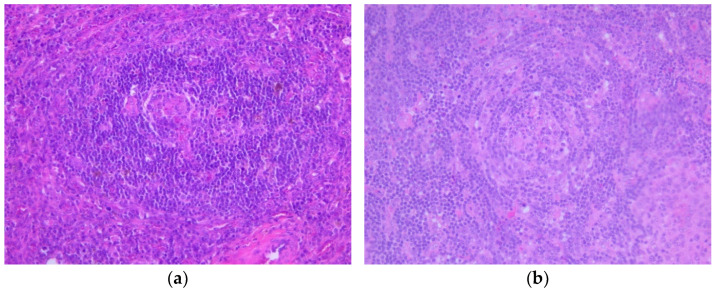

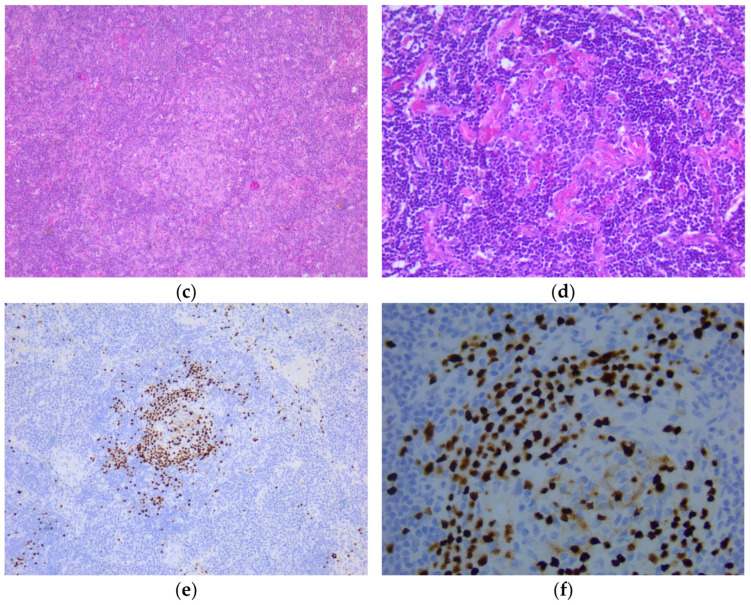
Microscopic aspects: (**a**) Atrophic germinal center, thickened mantle zone with a penetrating vessel in the center, ob. 10×; (**b**) Follicle with thickened mantle zone, lymphocytes arranged in layers, ob. 20× (**c**) Blurring boundaries of the mantle zone with fusion appearance of between adjacent germinal centers, ob. 10×; (**d**) vascular proliferation and hyalinization of vessel walls, ob. 20×; (**e**,**f**) HHV-8-LANA1, intense nuclear staining, ob. 10× and 40×.

**Figure 4:**
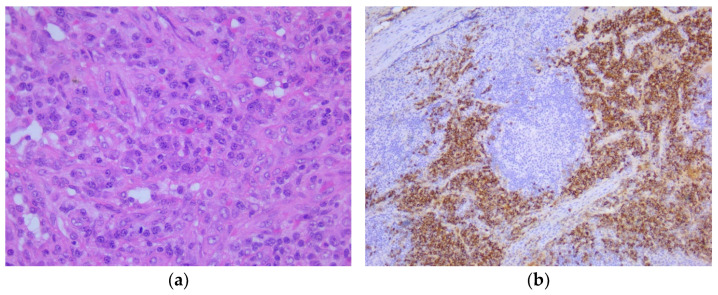

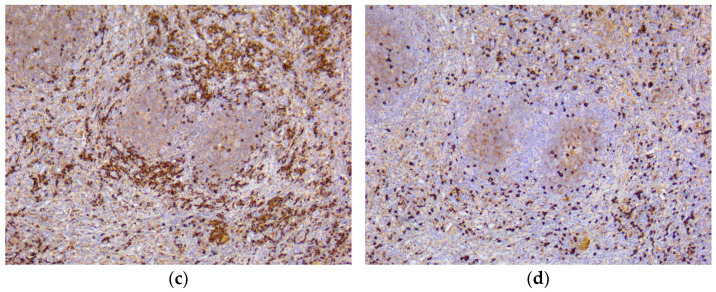
Microscopic aspects: (**a**) Hematoxylin–Eosin-stained slide showing plasma cells aggregate in the interfollicular area, ob. 40×; (**b**) CD138, positive reaction in plasma cells, highlighting the aggregates, ob. 10×; (**c**) Kappa light chain positive reaction, ob. 10×; (**d**) Lambda light chain positive reaction, ob. 10×.

**Table 1:** The patient’s laboratory results.

Laboratory Tests	Normal Values/Units	Patient’s Values
Red blood cells	4.7–6.1 × 10^6^/µL	2.71 × 10^6^/µL
Hemoglobin	13.5–17.5 g/dL	7.73 g/dL
Hematocrit	41–53%	22%
Mean Corpuscular Volume	80–100 fL	83.4 fL
Platelets	150–400 × 10^3^/µL	43.9 × 10^3^/µL
White blood cells	4–10 × 10^3^/µL	3.28 × 10^3^/µL
C-reactive protein	<5 mg/L	132.4 mg/L
Gamma globulin	12–22%	34.2%
Serum natrium	135–145 mmol/L	131 mmol/L
Procalcitonin	<0.05 ng/mL	10.1 ng/mL
IL-6	<7 pg/mL	122.7 pg/mL

**Table 2:** Data related to the antibodies used for immunohistochemical reactions.

Antibody	Substrate	Dilution	Clone
^1^ CD31	Monoclonal Mouse	1:100	JC70A
^2^ CD34	Monoclonal Mouse	1:100	QBEnd/10
^3^ D2-40	Monoclonal Mouse	1:100	322M-16
^4^ HHV-8-LANA1	Monoclonal Mouse	1:50	13B10
Kappa Light Chains	Monoclonal Mouse	1:200	CH15
Lambda Light Chains	Monoclonal Mouse	^5^ RTU	SHL53
^6^ CD138	Monoclonal Mouse	RTU	MI15
^7^ EBV	Monoclonal Mouse	RTU	CS1-4

^1^ CD31: Cluster of Differentiation 31; ^2^ CD34: Cluster of Differentiation 34; ^3^ D2-40: Podoplanin; ^4^ HHV-8-LANA1: Human Herpes Virus 8 Latency-Associated Nuclear Antigen 1; ^5^ RTU: ready-to-use; ^6^ CD138: Cluster of Differentiation 138; ^7^ EBV: Epstein–Barr virus.

**Table 3:** The patient’s laboratory results after treatment.

Laboratory Tests	Normal Values/Units	Patient’s Values
Red blood cells	4.7–6.1 × 10^6^/µL	4.37 × 10^6^/µL
Hemoglobin	13.5–17.5 g/dL	13.9 g/dL
Hematocrit	41–53%	41.1%
Mean Corpuscular Volume	80–100 fL	94.1 fL
Platelets	150–400 × 10^3^/µL	315 × 10^3^/µL
White blood cells	4–10 × 10^3^/µL	6 × 10^3^/µL
C-reactive protein	<5 mg/L	1.91 mg/L
Serum natrium	135–145 mmol/L	143 mmol/L
Procalcitonin	<0.05 ng/mL	0.06 ng/mL

## Data Availability

The data that support the fundings on this study are available from the corresponding author upon reasonable request.
